# A type-augmented knowledge graph embedding framework for knowledge graph completion

**DOI:** 10.1038/s41598-023-38857-5

**Published:** 2023-07-31

**Authors:** Peng He, Gang Zhou, Yao Yao, Zhe Wang, Hao Yang

**Affiliations:** 1 Zhengzhou University of Technology, Zhengzhou, China; 2 PLA Information Engineering University, Zhengzhou, China

**Keywords:** Engineering, Mathematics and computing, Nanoscience and technology

## Abstract

Knowledge graphs (KGs) are of great importance to many artificial intelligence applications, but they usually suffer from the incomplete problem. Knowledge graph embedding (KGE), which aims to represent entities and relations in low-dimensional continuous vector spaces, has been proved to be a promising approach for KG completion. Traditional KGE methods only concentrate on structured triples, while paying less attention to the type information of entities. In fact, incorporating entity types into embedding learning could further improve the performance of KG completion. To this end, we propose a universal Type-augmented Knowledge graph Embedding framework (TaKE) which could utilize type features to enhance any traditional KGE models. TaKE automatically captures type features under no explicit type information supervision. And by learning different type representations of each entity, TaKE could distinguish the diversity of types specific to distinct relations. We also design a new type-constrained negative sampling strategy to construct more effective negative samples for the training process. Extensive experiments on four datasets from three real-world KGs (Freebase, WordNet and YAGO) demonstrate the merits of our proposed framework. In particular, combining TaKE with the recent tensor factorization KGE model SimplE can achieve state-of-the-art performance on the KG completion task.

## Introduction

**Figure 1:**
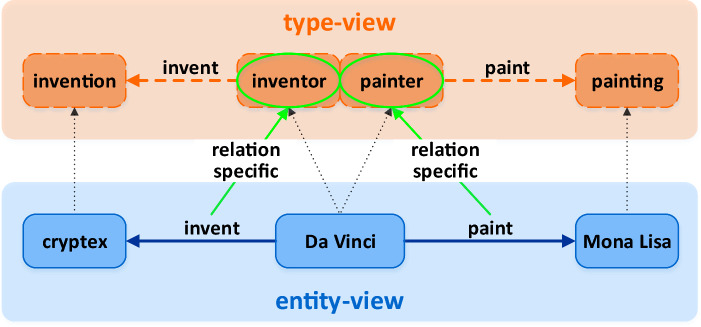
An example of a type-aware KG.

Knowledge graphs (KGs) collect and store human knowledge about the real world. A KG consists of a set of facts with the form of structured triples (*head, relation, tail*), in which *head* and *tail* are entities, *relation* is the semantic relationship from *head* to *tail*. Recently years, several large-scale KGs including Freebase^1^, WordNet^2^, YAGO^3^ and Google Knowledge Graph^4^ have been built and successfully used in a variety of artificial intelligence applications, such as question answering^5^, recommendation systems^6–8^, dialogue generation^9^ and natural language inference^10^. Although existing KGs contain a large number of factual triples, they still inevitably have the incomplete problem, which limits their usefulness in downstream applications. To address this problem, various approaches of KG completion (also known as link prediction) have been developed to auto-complete by predicting missing triples based on known ones. For example, in Fig. 1, knowing the head entity *Da Vinci* and the relation *paint*, the purpose of the KG completion task is to infer which of the existing entities in the KG is the missing tail entity, e.g., *Mona Lisa*. One of the most promising approaches for KG completion is knowledge graph embedding (KGE).

Inspired by the idea of distributed representation^11^, KGE aims to map the elements (entities and relations) of KG into continuous low-dimensional vector spaces to learn their distributed representations (a.k.a. embeddings) while retaining the inherent structure of KG. Because of its computational efficiency and compatibility with deep learning algorithms, KGE has been widely applied in various knowledge-driven tasks. The majority of existing KGE methods traditionally learn distributed representations only on the basis of single structured triples^12–21^, ignoring valuable additional information, e.g., entity types^22–24^, textual descriptions^25,26^ and temporal information^27–29^. Especially entity types, which provide the general semantics and categories of entities, could be intuitively incorporated into the process of KGE to guide distributed representation results and improve the performance of KG completion. As shown in Fig. 1, the relation *paint* always connects head entities of type *painter* to tail entities of type *painting*. Therefore, when we infer the missing tail entity of the triple (*Da Vinci, paint, ?*), we could utilize the prior knowledge of type constraint that the relation is *paint* and the head entity is *Da Vinci*, a *painter* to conclude the type of the tail entity should be a *painting*. Its position in the vector space should not be far away from other paintings, even though it has not sufficient known triples in training data for learning embeddings.

Several type-sensitive KGE models^22–24,30–33^, which try to take advantage of type information during embedding, have shown success. However, they are prone to one or more of the following drawbacks: (1) type information is tightly encoded into the optimization objective function, making the incorporation highly relevant to the training process and hence less flexible in extending new KGE models; (2) explicit type information is necessary, but in most real-world KGs, this type information is incomplete or even unavailable, which limits the versatility of models. Such as in FB15k^12^, 10% of entities with the */music/artist* type miss the */people/person* type^34^, and in WordNet, there is no type information available at all; (3) the diversity of entity types is neglected. However, in real-world KGs, an entity tends to belong to multiple types. More importantly, when associated with different relations, the entity may highlight distinct type features. As Fig. 1 shows, *Da Vinci* is a famous painter, and he is also an inventor, engineer, scientist, etc. When he is connected to *Mona Lisa* by the relation *paint*, he is classified as the type *painter*, and when he is connected to *cryptex* by the relation *invent*, he is classified as an *inventor*. It is a noteworthy problem since the ability to distinguish distinct type features focusing on different relations can obtain more refined type embeddings, which tune entity embeddings better.

To conquer the above drawbacks, we propose a Type-augmented Knowledge graph Embedding framework called TaKE, which can be combined with any traditional KGE model to enhance their ability of utilizing type information. As illustrated in Fig. 1, we first divide a type-aware KG into two views: the entity-view which consists of relation-entity triples connecting entities through relations, such as (*Da Vinci, paint, Mona Lisa*) and the type-view which contains the type information about entities, e.g., *Mona Lisa* is of type *painting*. It is obvious that semantic relations connecting entities can also connect types to which the entities belong. As a result, relation-entity triples in the entity-view (e.g., (*Da Vinci, paint, Mona Lisa*)) can be extended into the relation-type triples in the type-view (e.g., (*painter, paint, painting*)). Then, we map triples in different views into two distinct vector spaces. Intuitively, the higher-dimensional vector space represents more specific relation-entity triples, and the lower-dimensional vector space captures the general features of relation-type triples. Afterward, we design a type compatibility function to model the type constraint between entities and their connected relations, for learning implicit type features automatically without any explicit type information. Meanwhile, a relation-specific hyperplane mechanism is adopted to model the diversity of entity types. By projecting the type representation of an entity on different hyperplanes corresponding to its distinct connected relations, multiple type features of the entity can be highlighted and distinguished. Finally, we combine the type compatibility function and the score function of the chosen traditional KGE model to jointly measure the factuality of a given triple.

During the training process, we further use the type-constrained prior knowledge to construct a candidate set of homogeneous entities and a candidate set of non-homogeneous entities. Dynamic sampling is performed respectively from them, so as to provide high-quality negative samples and avoid insufficient negative samples due to data sparsity, making the training process more flexible and efficient.

In summary, the main work of this paper is as follows:

1. We propose a model-agnostic TaKE framework, which could be potentially combined with any traditional KGE models to extend it to be type-sensitive. Moreover, our framework can generalize well to all kinds of KGs because no explicit type information is required.
2. We also design a new type-constrained negative sampling strategy to construct more effective negative samples for training under no explicit type information supervision.
3. We combine TaKE with several existing traditional KGE models and evaluate them with the task of knowledge graph completion on four widely used benchmarks from three well-known KGs: Freebase, WordNet and YAGO. Experimental results demonstrate that TaKE-augmented KGE models consistently outperform their corresponding base models. Especially combining our framework with SimplE^20^, a recent tensor factorization KGE approach, can achieve the best KG completion performance compared with all baselines. Besides, we visualize the vectorial representations of types and entities to show that type embeddings can cluster better than entity embeddings, which validates TaKE dose capture type features well.

In the following part of this paper, we first provide some formal background and review the related work. Then, we introduce the details of our proposed framework and negative sampling strategy. The relevant explanation and analysis are also covered. Next, we report the experimental setup and results. Finally, we conclude the work together with possible future directions.

## Background and related work

Throughout the paper, we notate scalars using lower-case letters and vectors using bold lower-case letters. Such as, $\mathbf{{z}}\in \mathbb {R}^d$ is a real vector of length *d*, and $\mathbf{{z}}\in \mathbb {C}^d$ is a complex vector of length *d*. $\Vert \cdot \Vert _p$ denotes vectors’ *p*-norm, and $\langle \cdot \rangle$ denotes the sum of Hadamard product (element-wise product) of vectors.

**Knowledge graph (completion):** A knowledge graph (KG) can be viewed as a multi-relational directed graph *G*, which is organized and stored in the form of factual triples $G=\{{E},{R},{T}\}$. Among them, *E* represents a set of nodes, *R* represents a set of edges, and ${T}=\{(h,r,t)\vert h,t \in {E};r \in {R}\} \subseteq {E}\times {R}\times {E}$ represents a set of factual triples, where *h*, *t* are the head entity and tail entity respectively, *r* is the semantic relation from *h* to *t*. These factual triples capture human knowledge of the real world. KG completion is a task of inferring all factual triples *W* based on the known *T*.

**(Type-sensitive) Knowledge graph embedding:** Knowledge graph embedding (KGE) is also called knowledge graph representation learning (KGRL), which aims to learn distributed representations (vectors, matrices, or tensors) of elements in KGs. The KGE process usually consists of four steps: (1) define an embedding function to map all known entities and relations into vector spaces; (2) define a score function $f(\cdot )$. For a given triple (*h*, *r*, *t*), the input of this score function is the embedding representations of *h*, *t* and *r* from step (1), and the output is a scalar score representing the factuality of the triple. The higher score demonstrates the more factual of the triple; (3) design a negative sampling strategy to generate negative samples based on known triples from the input KG; (4) optimize a suitable loss function to learn the values of embeddings using both known and negative triples. Type-sensitive KGE is to integrate explicit or implicit type information during representation learning.

During the past decade, a variety of KGE models have been widely explored for the KG completion task. In the following, we will introduce three lines of them that are closely related to our work. For further details on these methods, please refer to recent surveys^35–37^.

### KGE based on single triples

Most of the existing KGE models rely on single triples to learn vectorial embeddings, which just exploit the structured information implied in KGs. We call this kind of KGE model the traditional KGE model. According to their score functions, these traditional KGE models can be generally classified into two categories, one is based on the translation and the other is inspired by tensor factorization techniques.

TransE^12^ is a typical translation-based model. It defines an embedding function to embed entities and relations into the same real vector space, i.e., $\mathbf{{h}},\mathbf{{r}},\mathbf{{t}}\in \mathbb {R}^d$. For each triple (*h*, *r*, *t*), TransE regards the relation *r* as a translation operation from the head *h* to the tail *t* in the vector space and employs the distance between the translated head entity and tail entity as a metric to measure the authenticity of this triple. Thus the score function of TransE is

1 $$\begin{aligned} \hfil f(h,r,t)=- {\Vert \mathbf{{h}} + \mathbf{{r}} - \mathbf{{t}} \Vert _{1/2}}. \end{aligned}$$

Although simple and easy to understand, TransE has difficulty in modeling common symmetric relations as well as complex one-to-many, many-to-one and many-to-many relations. To overcome these problems, many variants of TransE have been developed. Such as, TransH^13^ projects entity embeddings on different relation-specific hyperplanes and regards relations as translation operations between projected entity embeddings. Instead of relation-specific hyperplanes, TransR^14^ directly projects entity embeddings from the entity space to the relation-specific space through transformation matrices and judges the translated distance between entities in the relation-specific space. More recently, Sun et al.^15^ found that the success of a KGE model heavily relies on its ability to model common relation patterns in KGs including symmetry, antisymmetry, inversion, and composition. For modeling and inferring these relation patterns, they proposed RotatE which maps triples to complex vector spaces instead of real vector spaces. Moreover, RotatE interprets each relation as a rotation operation from its connected head embedding to tail embedding. Soon after RotatE, HAKE^16^ and RatE^17^ make slight improvements to it. By taking full advantage of both the angular and modulus parts of complex vectors, HAKE can not only infer four common relation patterns mentioned above but also model semantic hierarchies between entities. While RatE promotes the expressive power of RotatE and handles one-to-many relations effectively via a learnable relation-specific weighted product.

Apart from translation-based models, tensor factorization models are also competitive on many benchmarks, including DistMult^18^, ComplEx^19^, SimplE^20^, etc. DistMult defines the same embedding function as in TransE, but the score function is the sum of Hadamard product of embedding vectors:

2 $$\begin{aligned} \hfil f(h,r,t)=\langle \mathbf{{h}},\mathbf{{r}},\mathbf{{t}} \rangle . \end{aligned}$$

Since DistMult constrains the tensor in the head and tail modes to be symmetric to keep the parameter sharing scheme, it can only model the symmetric relation pattern. To overcome this shortcoming, ComplEx enhances DistMult by mapping elements into a complex space, where head and tail embeddings share the parameters of values but are complex conjugates to each other. SimplE is another tensor factorization approach based on Canonical Polyadic (CP) decomposition^38^, which employs inverse relations to associate two embeddings of the same entity locating at different positions. Both ComplEx and SimplE are fully expressive and universally applicable to all kinds of downstream tasks^20^. But compared with ComplEx, SimplE avoids computational redundancy and reduces time consumption. Lacroix et al.^21^ also propose a CP decomposition-based model similar to SimplE.

Although traditional models have achieved success, their representing and modeling capabilities are still limited due to the single structured information. Therefore, all kinds of extensions of traditional KGE models have been developed through integrating rich additional information into the process of representation learning, including entity types, lexical descriptions, temporal information, and so on. Compared with other additional information, type information is simpler and contains less noise, so it is conceivable to incorporate type information to improve the performance of KGE.

### KGE incorporating type information

There have been several type-sensitive KGE models exploring the usage of type information. TKRL^22^ extends traditional TransE by explicitly introducing type information. It maps hierarchical types as projection matrices of entities to make entities have distinct representations in different types. TransC^23^ clearly distinguish concepts (i.e. entity types) and instances (i.e. entities). In order to capture the semantic transitivity between concepts and instances as well as concepts at distinct levels, TransC encodes the concept as a sphere, the instance as a vector, and uses their relative positions to model the semantic transitivity. JOIE^24^ directly represents a KG as two views: ontology view and instance view, and jointly encodes these two views. Both TransT^30^ and TaRP^32^ collect relation types from entity types and compute prior probabilities to indicate the semantic similarity of relation types and entity types based on Bayes’ rule. TaRP extends TransT to further consider underlying hierarchy structures among types when estimating the prior probability. Besides, Niu et al.^33^ propose CAKE which automatically extract commonsense from factual triples with type information.

All of the above models expect explicit supervision from type information. In view of the fact that some real-world KGs lack or even have no this information (e.g., Freebase and WordNet), Jain et al.^31^ propose TypeDM and TypeComplex to enhance DistMult and ComplEx respectively by modeling type constraint between relations and associated entities. Although TypeDM and TypeComplex learn implicit type features in entities automatically without any explicit type information. The learning and utilizing of type features are highly relevant to specific score functions and loss functions. Therefore, they are difficult to apply to other traditional models, limiting their modeling capability and universal applicability to downstream tasks^20^.

Comparably, our proposed TaKE framework can be combined with any traditional KGE models flexibly to enhance their ability of exploiting type information, by capturing and integrating implicit type features under no explicit type information supervision. Especially, when TaKE is combined with fully expressive traditional models, such as SimplE, the TaKE-augmented models are also fully expressive. Moreover, during learning type features automatically, TaKE models both type constraint and type diversity with low time and space complexity.

### Negative sampling strategy in KGE

Conducting negative sampling based on known positive triples is important in KGE, but how to construct effective negative samples is still a challenging problem. Most of the existing KEG models carry out the uniform sampling scheme as in^12^. To generate corrupted triples, this scheme samples a random entity uniformly from all known entities to replace either the head entity or the tail entity of each positive triple. However, this uniform sampling scheme tends to introduce false negative samples. In order to reduce false negative samples, Wang et al.^13^ define a Bernoulli distribution to replace the head and tail entities with different probabilities. Specifically, it gives more chance to replace the head entity if the relation is one-to-many and gives more chance to replace the tail entity if the relation is many-to-one. Recently, Sun et al.^15^ propose a self-adversarial negative sampling strategy and design a self-adversarial negative sampling loss as the optimization object.

Other works attempt to employ prior knowledge in the form of type constraint to generate more competitive negative samplings. Type-constrained method^39^ applies a local closed-world assumption based on observed triples. For a given positive triple, it only uses the head or tail entities that have appeared at the same relation to corrupt the head or tail entities. However, this hard constraint of entity types tends to hinder the normal clustering of similar entities and lose the information of other possible candidate entities. Considering this, TKRL proposes an improved negative sampling strategy named Soft Type Constraint (STC). It selects entities in the same type with a bigger probability than others. But the sampling probability is a fixed constant, lacking flexibility, and same as its model TKRL, STC requires explicit type information available.

To deal with the above issues, we design a new type-constrained negative sampling strategy in which explicit type information is not necessary. We not only construct a candidate set of homogeneous entities based on the local closed-world assumption, but also construct a candidate set of non-homogeneous entities to maintain normal clustering of homogeneous entities while avoiding the information loss of non-homogeneous entities. Moreover, our strategy dynamically samples from both candidate sets according to the current state of the model to further improve training efficiency.

## Proposed framework

**Figure 2:**
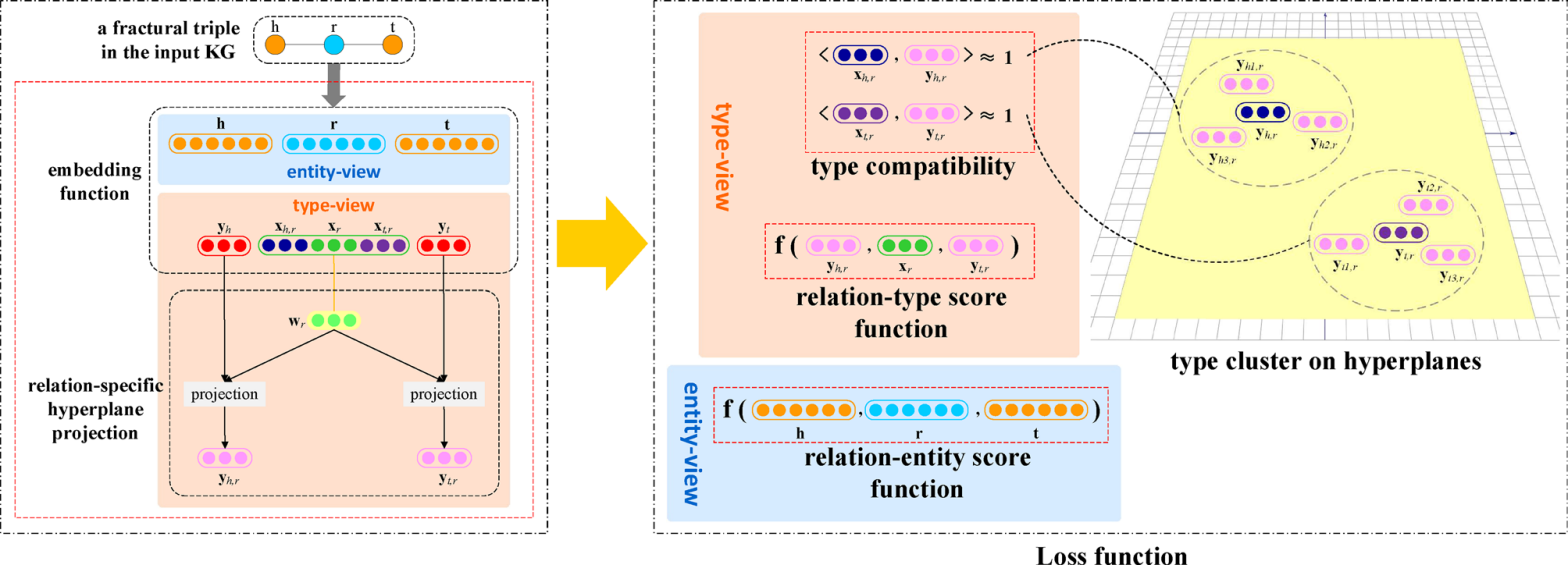
The architecture of TaKE.

In this part, we introduce our framework TaKE which aims to construct type-sensitive versions of TransE, DistMult, ComplEx, SimplE, RotatE, or any other traditional model via learning type representations of entities. And we refer to these type-sensitive models as TaKE-augmented models, such as TaKE-TransE, TaKE-DistMult, TaKE-ComplEx, TaKE-SimplE and TaKE-RotatE. As Fig. 2 shows, we first adopt the embedding function of the chosen traditional model to map the elements of the input KG into two vector spaces of different dimensions, which represent the semantics of relation-entity triples and relation-type triples. Then, type constraint is modeled by designing a type compatibility function based on semantic similarity to learn and constrain type features of entities. At the same time, we further distinguish multiple types of the same entity via a relation-specific hyperplane projection mechanism. Afterward, the factuality of relation-type triples and relation-entity triples is measured on the basis of the score function of the chosen traditional model. And the overall score function is a combination of both the type compatibility function and score functions. Finally, we describe our designed type-constrained negative sampling strategy and the loss function in detail.

### Embedding function

In order to capture type features implied in known entities and relation-entity triples, we regard the factual triples from input KGs as an entity-view. Based on this entity-view, we introduce a type-view containing relation-type triples. In these triples, relations are from semantic relations connecting entities in the entity-view, and types connected by relations are what we expect to learn automatically. Intuitively, the more specific entity-view should be embedded in a higher-dimensional vector space and the more general type-view should be embedded in a lower-dimensional vector space.

We represent relation-entity triples in entity-view into a vector space with dimension *d* and relation-type triples in type-view into a vector space with dimension *k*. Typically, *k* is much smaller than *d* ($k \ll d$), and whether it is a real vector space $\mathbb {R}$ or a complex vector space $\mathbb {C}$ is dependent on which existing KGE model our framework will be combined with. Taking the TaKE-DistMult for example, the embedding function of DistMult is defined to map elements of the input KG into a real vector space. So in our case, each entity $e \in \{h,t\}$ is mapped into two real vectors: $\mathbf{{e}}\in \mathbb {R}^d$ and $\mathbf{{y}}_e \in \mathbb {R}^k$. Among them, $\textbf{e}$ encodes individual features of entities, and $\mathbf{{y}}_e$ encodes general type features of entities. Similarly, each relation *r* is also mapped into two real vectors: $\mathbf{{r}}\in \mathbb {R}^d,\mathbf{{x}}_r\in \mathbb {R}^k$, where $\mathbf{{r}}$ denotes the semantic information of the relation when it connects two entities in entity-view, and $\mathbf{{x}}_r$ denotes the semantic information of the relation when it connects two entity types in type-view.

Besides, for modeling type constraint between entities and relations, we define the head or tail relation type as the head or tail entity type that the relation expects to connect. And we add two type embeddings $\mathbf{{x}}_{h,r}, \mathbf{{x}}_{t,r}\in \mathbb {R}^k$ for each relation to represent the head relation type and the tail relation type.

### Type compatibility function and relation-specific hyperplane projection

In KGs, there exist type constraint between entities and relations. That is, for a given triple (*h*, *r*, *t*), the relation *r* always connects the head entity *h* and the tail entity *t* categorized as the same type. As Fig. 1 shows in the introduction, the relation *paint* always connects head entities of type *painter* (e.g., *Da Vinci*) and tail entities of type *painting* (e.g., *Mona Lisa*). Because only painters could paint paintings. If the head entity of relation *paint* is not a painter or the tail entity is not a painting, it must be a false triple and cannot exist in KGs. In order to capture this type constraint specific to relations, we design a type compatibility function to constrain type embeddings of entities and relations. And this type compatibility function is based on semantic similarity of type features. Therefore, the type compatibility functions of the head relation type $\mathbf{{x}}_{h,r}$ and the head entity type $\mathbf{{y}}_{h}$, as well as the tail relation type $\mathbf{{x}}_{t,r}$ and the tail entity type $\mathbf{{y}}_{t}$ are:

3 $$\begin{aligned} \hfil f_{1}(h,r)= & {} \sigma \langle \mathbf{{x}}_{h,r},\mathbf{{y}}_{h} \rangle , \end{aligned}$$

4 $$\begin{aligned} \hfil f_{1}(t,r)= & {} \sigma \langle \mathbf{{x}}_{t,r},\mathbf{{y}}_{t} \rangle . \end{aligned}$$

where $\sigma$ is a nonlinear operation (e.g., sigmoid). If the triple is positive, the values of these two type compatibility functions are expected to be almost 1, otherwise close to 0. Take type compatibility function (3) for example. Since the embedding $\mathbf{{x}}_{h,r}$ represents the head entity type that relation *r* expects to connect with, and the embedding $\mathbf{{y}}_{h}$ represents the type feature of head entity *h*. For positive triples, because of relation-specific type constraints, the values of $\mathbf{{x}}_{h,r}$ and $\mathbf{{y}}_{h}$ should be as similar as possible. That is, the value of $f_{1}(h,r)$ should be almost 1. For negative triples, the values of $\mathbf{{x}}_{h,r}$ and $\mathbf{{y}}_{h}$ should be as different as possible. That is, the value of $f_{1}(h,r)$ should be almost 0. By optimizing the final loss function containing $f_{1}(h,r)$, the framework can learn the type feature $\mathbf{{y}}_{h}$ of head entity *h* automatically under no explicit type information.

However, the type embeddings learned above are coarse-grained entity type features. Moreover, in real-world KGs, an entity tends to highlight distinct type features when it is connected to different relations. As Fig. 1 shows in the introduction, when *Da Vinci* is connected to the relation *paint*, he highlights the *painter* type. And when he is connected to the relation *invent*, he emphasizes on the *inventor* type. In order to further distinguish and learn more-refined entity type features specific to different relations, we introduce a hyperplane projection mechanism to model type diversity. Specifically, we segregate the embedding space of type-view into different zones with the help of hyperplanes. Each relation *r* in the KG is associated with a hyperplane represented by a normal vector $\mathbf{{w}}_r\in \mathbb {R}^k, \Vert \mathbf{{w}}_r \Vert _2=1$. Thus, we get $\vert {R}\vert$ relation-specific hyperplanes and $\vert {R}\vert$ is the number of relations. If an entity $e \in \{h,t\}$is connected to one or more relations, e.g., $r_i,r_j,\ldots$, its type embedding $\mathbf{{y}}_e$ learned through the type compatibility functions (3) and (4) is projected on corresponding hyperplanes of these connected relations $\mathbf{{w}}_{r_i},\mathbf{{w}}_{r_j},\ldots$:

5 $$\begin{aligned} \hfil \mathbf{{y}}_{e,r_i}=\mathbf{{y}}_e-\mathbf{{w}}_{r_i}^\top \mathbf{{y}}_e\mathbf{{w}}_{r_i}, \quad \mathbf{{y}}_{e,r_j}=\mathbf{{y}}_e-\mathbf{{w}}_{r_j}^\top \mathbf{{y}}_e\mathbf{{w}}_{r_j}, \ldots \end{aligned}$$

where $\mathbf{{y}}_{e,r_i}$ and $\mathbf{{y}}_{e,r_j}$ represent relation-specific type embeddings. In this way, the type embedding of an entity connected by different relations could be projected to distinct relation-specific hyperplanes. Therefore, multiple types of entities could be distinguished according to different associated relations. Such as, by projecting the type embedding of entity *Da Vinci* obtained from type compatibility functions onto hyperplanes specific to relations *paint* and *invent* respectively, it’s possible to distinguish two type features *painter* and *inventor* implied in the entity.

With these more-refined type features, the above type compatibility functions become:

6 $$\begin{aligned} \hfil f_{1}(h,r)= & {} \sigma \langle \mathbf{{x}}_{h,r},\mathbf{{y}}_{h,r} \rangle , \end{aligned}$$

7 $$\begin{aligned} \hfil f_{1}(t,r)= & {} \sigma \langle \mathbf{{x}}_{t,r},\mathbf{{y}}_{t,r} \rangle . \end{aligned}$$

where $\mathbf{{y}}_{h,r}$ and $\mathbf{{y}}_{t,r}$ are the head entity type specific to relation *r* and the tail entity type specific to relation *r* respectively.

### Score function

TaKE-DistMult utilizes the score function of DistMult to evaluate whether the relation-entity triples and relation-type triples exist or not. Since the score function of DistMult is the dot product of vectorial embeddings, the value range of this score function is from 0 to 1. As a result, we get the score function for relation-entity triples:

8 $$\begin{aligned} \hfil f_{2}(h,r,t)= \langle \mathbf{{h}},\mathbf{{r}},\mathbf{{t}}\rangle , \end{aligned}$$

and the score function for relation-type triples:

9 $$\begin{aligned} \hfil f_{3}(h,r,t)= \langle \mathbf{{y}}_{h,r},\mathbf{{x}}_r,\mathbf{{y}}_{t,r}\rangle . \end{aligned}$$

Finally, the overall score function of TaKE-DistMult for the given triple (*h*, *r*, *t*) is a combination of two type compatibility functions (6)(7) and two score functions (8)(9):

10 $$\begin{aligned} \hfil f(h,r,t) = f_{1}(h,r)f_{1}(t,r)f_{2}(h,r,t)f_{3}(h,r,t). \end{aligned}$$

### Negative sampling strategy and loss function

For learning knowledge more efficiently in the training phase, we design a new type-constrained negative sampling strategy without requiring any explicit type information. We first construct a set of homogeneous entities and a set of non-homogeneous entities. Then, our strategy selects corrupting entities from the set of homogeneous entities and the set of non-homogeneous entities respectively according to a specific proportion. Meanwhile, this proportion is dynamically updated by the current model.

We first introduce the formal description of the entities set. Based on the local closed-world assumption^39^, for a positive triple (*h*, *r*, *t*) from the input KG, the homogeneous entity set used to corrupt the head entity *h* is

11 $$\begin{aligned} \hfil {E}_t=\{h' \in {E} \vert (h',r,t) \notin {T} \wedge \exists e \in {E}:(h',r,e) \in {T} \}. \end{aligned}$$

That is, we introduce prior knowledge that only relation-specific entities can be chosen to construct negative triples. Such as the fact (*Zooey Claire Deschanel, act in TV, New Girl*) extracted from Freebase, we could construct a negative sample (*June Allyson, act in TV, New Girl*) by corrupting the head entity *Zooey Claire Deschanel*, but never (*Andrew Stanton, act in TV, New Girl*). Since *June Allyson* is also an actress, but she did not act in *New Girl*, while *Andrew Stanton* never appeared in the head position of the relation *act in TV*. However, only sampling from the homogeneous entities tends to hinder the normal clustering of homogeneous entities. And because of graph sparseness, homogeneous entities appearing in the head (or tail) position of a relation are usually limited. Thus, we may not obtain enough negative samples. On the other hand, although *Andrew Stanton* is not an actor, he is a director that tends to be close to actors in embedding spaces. Therefore, rejecting the possible negative sample (*Andrew Stanton, act in TV, New Girl*) will result in a loss of information.

In order to conquer these drawbacks, we further introduce the non-homogeneous entity set denoted as: $\bar{{E}}_t={E}-{E}_t-h$. It is noteworthy that although we only present the candidate entity set of corrupting head entities, the candidate entity set of corrupting tail entities is also considered in our implementation.

For each positive triple, the probability of selecting entities from the homogeneous entity set to construct negative samples is as follows:

12 $$\begin{aligned} \hfil P(h' \in {{E}_t}) = \frac{{k\vert {{E}_t}\vert }}{{\vert {\bar{{E}}_t}\vert + k\vert {{E}_t}\vert }}, \end{aligned}$$

where $\vert {{E}_t}\vert$ and $\vert {\bar{{E}}_t}\vert$ are the entity numbers of ${E}_t$ and $\bar{{E}}_t$ respectively. *k* is a hyper-parameter indicating that the probability of selecting entities from ${E}_t$ is *k* times bigger than the probability of selecting entities from $\bar{{E}}_t$. We initialize *k* as 1 and updated dynamically according to the current model during training:

13 $$\begin{aligned} \hfil k \leftarrow \frac{1}{\vert {T} \vert }\sum \nolimits _{T} {\frac{\sum \nolimits _{(h',r,t) \in {N}} \exp f(h',r,t) /\vert {N} \vert }{\sum \nolimits _{(h',r,t) \in \bar{{N}}} \exp f(h',r,t) /\vert \bar{{N}}\vert }}, \end{aligned}$$

where *N* represents the set of negative samples constructed by replacing head entities with candidate entities in ${E}_t$, and ${\bar{{N}}}$ represents the set of negative samples constructed by replacing head entities with candidate entities in $\bar{{E}}_t$. Thus, *k* tends to the set containing more challenging negative samples and the effect of the current model is weakened by prior knowledge.

Since the cross entropy loss function has shown good performance in existing KGE models^20,21,28^, we optimize the regularized cross entropy loss function:

14 $$\begin{aligned} \hfil L= \frac{1}{\vert {T} \vert }\sum \nolimits _{(h,r,t) \in {T}} \Big [-f(h,r,t) + \log \Big ({\sum \nolimits _{(h',r,t) \in {N} \cup \bar{{N}}} {\exp (f(h',r,t))} } \Big ) + \lambda \Omega ^p \Big ], \end{aligned}$$

where

15 $$\begin{aligned} \hfil \Omega ^p= \frac{1}{p} \big ( \Vert \mathbf{{h}}\Vert _p^p + \Vert \mathbf{{t}}\Vert _p^p + \Vert \mathbf{{r}}\Vert _p^p + \Vert \mathbf{{y}}_h\Vert _p^p + \Vert \mathbf{{y}}_t\Vert _p^p + \Vert \mathbf{{x}}_{h,r}\Vert _p^p + \Vert \mathbf{{x}}_{t,r}\Vert _p^p \big ) \end{aligned}$$

is the p-norm regularizer and $\lambda$ is a weighted hyperparameter. Usually, we set $p=2$ to get a squared Frobenius norm regularizer.

### Time and space complexity

As described in^40^, to scale to the size of current KGs and keep up with their growth, a KGE embedding model must have a linear time and space complexity. Models with a large number of parameters tend to overfit and present poor scalability. We calculate and analyze the time complexity and the number of parameters for existing type-sensitive KGE approaches TypeDM and TypeComplex, as well as TaKE-augmented models including TaKE-TransE, TaKE-DistMult, TaKE-ComplEx, TaKE-SimplE and TaKE-RotatE. They are listed in Table 1, where *d* is the dimension of the embedding vectors to represent entities and *k* is the dimension of the embedding vectors for entity types. From this table, we can conclude that compared with TypeDM and TypeComplex, TaKE-augmented models also have the linear time complexity $O(d+k)$. But in the number of parameters, TaKE-augmented models generally need a little more. However, since *k* is usually much smaller than *d*, this increase of parameters is negligible, especially compared with the better performance TypeComplex. Besides, TaKE-augmented models could distinguish diverse entity types specific to different relations and achieve superior performance in KG completion than TypeDM and TypeComplex.

**Table 1:** Time complexity and number of parameters for each model considered.

Model	Time complexity	Number of parameters
TypeDM	$O(d+k)$	$d(\vert {E}\vert +\vert {R}\vert )+k(\vert {E}\vert +2\vert {R}\vert )$
TypeComplex	$O(d+k)$	$d(2\vert {E}\vert +2\vert {R}\vert )+k(2\vert {E}\vert +4\vert {R}\vert )$
TaKE+TransE	$O(d+k)$	$d(\vert {E}\vert +\vert {R}\vert )+k(\vert {E}\vert +4\vert {R}\vert )$
TaKE+Distmult	$O(d+k)$	$d(\vert {E}\vert +\vert {R}\vert )+k(\vert {E}\vert +4\vert {R}\vert )$
TaKE+ComplEx	$O(d+k)$	$d(2\vert {E}\vert +2\vert {R}\vert )+k(2\vert {E}\vert +7\vert {R}\vert )$
TaKE+SimplE	$O(d+k)$	$d(2\vert {E}\vert +2\vert {R}\vert )+k(2\vert {E}\vert +7\vert {R}\vert )$
TaKE+RotatE	$O(d+k)$	$d(2\vert {E}\vert +2\vert {R}\vert )+k(2\vert {E}\vert +7\vert {R}\vert )$

## Experiments and results

In this section, we evaluate the effectiveness of TaKE to improve the performance of existing KGE models on KG completion. Three type-sensitive KGE models are also compared as baselines. Then we perform ablation studies to empirically verify the new type-constrained negative sampling strategy and relation-specific hyperplane projection mechanism are effective. Afterward, entity and type embeddings are clustered and visualized to demonstrate the ability of TaKE to capture type features. In the end, we empirically analyze and compare the time efficiency.

**Datasets:** Our datasets are four real-world benchmarks that have been widely used for KG completion: FB15K^12^, FB15K-237^41^, WN18^12^ and YAGO3-10^42^. FB15K and FB15K-237 are two subsets extracted from the common KG Freebase. To avoid the test set leakage problem pointed out by^41,42^, FB15K-237 removes all inverse relations from FB15K. WN18 is a subset of WordNet and YAGO3-10 is a subset of YAGO. Following^12^, we split each dataset into *training*, *validation* and *test* sets. Table 2 lists the statistics of four datasets.

**Table 2:** Statistics of four datasets.

Dataset	$\vert {E} \vert$	$\vert {R}\vert$	$\vert training \vert$	$\vert validation \vert$	$\vert test \vert$
FB15K	14,951	1,345	483,142	50,000	59,071
FB15K-237	14,541	237	272,115	17,535	20,466
WN18	40,943	18	141,442	5,000	5,000
YAGO3-10	123,182	37	1,079,040	5,000	5,000

**Evaluation metrics:** We choose two widely used metrics to evaluate the performance of link prediction. One is *Mean Reciprocal Rank (MRR)*, and the other is *Hits@N*. In order to introduce these two metrics, we first create two candidate sets $(h',r,t)$ and $(h,r,t')$ for each triple (*h*, *r*, *t*) in the *test* set. Specifically, the head *h* and the tail *t* of each test triple (*h*, *r*, *t*) are replaced by each known entity $e\in {E}$ in turn. Then we use the score function to calculate the scores for test triples and candidate triples, and rank them in descending order according to their scores. We evaluate them in the filtered setting as in^12^. That is, candidate triples that have appeared in the *training* set or *validation* set are ignored. *MRR* is the average of the reciprocal rankings of all test triples:

16 $$\begin{aligned} \hfil MRR = \frac{1}{{2\vert test \vert }}\sum _{(h,r,t) \in test} {\left( \frac{1}{{ran{k_h}}} + \frac{1}{{ran{k_t}}}\right) }, \end{aligned}$$

where $ran{k_h}$ and $ran{k_t}$ represent the rankings of each test triple in its corresponding candidate sets. Compared to another similar metric *Mean Rank (MR)*, which is largely influenced by a single bad prediction, *MRR* is more stable^43^. *Hits@N* exhibits the proportion of test triples ranked in the top *N*, which is defined as

17 $$\begin{aligned} \hfil Hits@N = \frac{1}{{2\vert test \vert }}\sum _{(h,r,t) \in test} [C(ran{k_h} \le N) + C(ran{k_t} \le N)], \end{aligned}$$

where *C*(*x*) is 1 if *x* holds and 0 otherwise. *MRR* and *Hits@N* have been standard evaluation measures for the KG completion task^15,19,20,24,28^ and higher *MRR* and *Hits@N* indicate better possible performance.

**Baselines:** We compare TaKE-augmented models with their base models including TransE, DistMult, ComplEx, SimplE and RotatE. We also consider type-sensitive KGE models TypeDM and TypeComplex as baselines, which learn entity type features during representation learning without explicit type information supervision. All the baselines have shown good performance on KG completion, and source codes of them are provided for the reproducibility of the results.

**Implementation:** For the results of baselines on datasets, we use the released codes and follow the similar experimental setups as in^20,31^ to ensure the fairness of the results. We implement our framework and TaKE-augmented models in PyTorch^44^. Adam^45^ are used as the optimizer and we validate every 20 epochs to select the optimal parameters giving the best validation *MRR*. The maximum number of iterations is set to 1000 and the batch size $b=4000$ is taken for all benchmarks. The ranges of other hyperparameters for grid search are set as follows: embedding dimensionality of entity-view $d\in \{100,200,500\}$, embedding dimensionality of type-view $k\in \{10,20,50\}$, weighted hyperparameters for the regularizer $\lambda \in \{0.1,0.3,1.0\}$, learning rate used for SGD $lr\in \{0.1,0.5,1.0\}$. The best configurations for all TaKE-augmented models are as follows: $d=200, k=20, \lambda =0.3, lr=0.5$ on FB15K and FB15K-237; $d=200, k=20, \lambda =0.3, lr=0.1$ on WN18; $d=500, k=20, \lambda =0.3, lr=0.5$ on YAGO3-10.

### Link prediction

We show the link prediction performance of TaKE-augmented models against their base models: TransE, DistMult, ComplEx, SimplE and RotatE as well as two recent type-sensitive models: TypeDM and TypeComplex. Tables 3 and 4 list the experimental results on four datasets. From these results, we have the following observations.

**Table 3:** Link prediction results on FB15K and FB15K-237. The best results are in bold.

Model	FB15K				FB15K-237			
	*MRR*	*Hits@1*	*Hits@3*	*Hits@10*	*MRR*	*Hits@1*	*Hits@3*	*Hits@10*
TransE	0.380	0.231	0.478	0.649	0.294	–	–	0.465
TaKE-TransE	0.490	0.365	0.574	0.699	0.301	0.199	0.337	0.486
DistMult	0.654	0.546	0.733	0.824	0.241	0.155	0.263	0.419
TaKE-DistMult	0.757	0.674	0.823	0.890	0.259	0.178	0.287	0.432
ComplEx	0.692	0.599	0.759	0.840	0.247	0.158	0.275	0.428
TaKE-ComplEx	0.778	0.701	0.839	0.895	0.267	0.189	0.206	0.442
SimplE	0.727	0.660	0.773	0.838	0.309	0.228	0.340	0.476
TaKE-SimplE	**0.806**	0.751	**0.842**	0.897	**0.352**	**0.255**	**0.390**	**0.549**
RotatE	0.797	0.746	0.830	0.884	0.338	0.241	0.375	0.533
TaKE-RotatE	0.804	**0.752**	0.838	**0.902**	0.347	0.252	0.386	0.541
TypeDM	0.750	0.673	–	0.861	0.252	0.178	–	0.425
TypeComplex	0.761	0.688	–	0.867	0.259	0.186	–	0.431

**Table 4:** Link prediction results on WN18 and YAGO3-10. The best results are in bold.

Model	WN18				YAGO3-10			
	*MRR*	*Hits@1*	*Hits@3*	*Hits@10*	*MRR*	*Hits@1*	*Hits@3*	*Hits@10*
TransE	0.495	0.113	0.888	0.943	0.18	–	–	0.37
TaKE-TransE	0.565	0.218	0.930	0.951	0.327	0.279	0.375	0.488
DistMult	0.797	–	–	0.946	0.34	0.24	0.38	0.54
TaKE-DistMult	0.930	0.826	0.904	0.952	0.410	0.321	0.450	0.608
ComplEx	0.941	0.936	0.945	0.947	0.36	0.26	0.40	0.55
TaKE-ComplEx	0.950	0.949	0.954	0.951	0.428	0.330	0.467	0.618
SimplE	0.942	0.939	0.944	0.947	0.488	0.395	0.543	0.645
TaKE-SimplE	**0.956**	**0.954**	**0.957**	**0.961**	**0.556**	**0.472**	0.610	**0.705**
RotatE	0.949	0.944	0.952	0.959	0.495	0.402	0.550	0.670
TaKE-RotatE	0.953	0.949	0.955	0.958	0.552	0.467	**0.611**	0.701
TypeDM	0.926	0.824	0.900	0.948	0.406	0.311	–	0.606
TypeComplex	0.939	0.932	–	0.951	0.411	0.319	–	0.609

First and foremost, TaKE-augmented models outperform their base models in *MRR* and *Hits@1/3/10* in most cases on all of the four datasets. Although on the WN18 dataset, RotatE’s *Hits@10* has slightly better performance, but the results are comparable. This observation provides evidence for the merit of capturing type features. Besides, the observation that TaKE-augmented models improve the performance of both translation-based models (TransE, RotatE) and tensor factorization models (DistMult, ComplEx, SimplE) indicates the effectiveness of TaKE framework, and also shows the potential of applying the framework on other KGE models.

On the other hand, TaKE-augmented models beat two type-sensitive methods on all datasets. Especially TaKE-SimplE and TaKE-RotatE give best results. Specifically, as the type extension of DistMult and ComplEx, TaKE-DistMult and TaKE-ComplEx significantly outperform TypeDM and TypeComplex on all datasets. Since TaKE-DistMult and TaKE-ComplEx could model the diversity of entity types specific to different relations, while TypeDM and TypeComplex do not consider this diversity of types. Besides, TypeDM and TypeComplex don’t exploit type information at the negative sampling stage. The superior performance of TaKE-augmented models empirically shows the importance of modeling the diversity of types and the exploiting type information to generate negative samples in the link prediction process.

**Figure 3:**
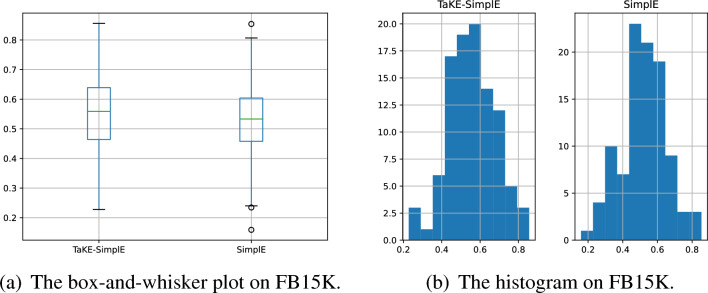
The statistical results of TaKE-SimplE and SimplE for link predictions on FB15K.

In order to further demonstrate the reliability of the above experimental results, we conduct the significant test in Tables 3 and 4. Specifically, we choose TaKE-SimplE and SimplE to train on FB15K and implement 100 times independent link prediction experiments. Each time, we set different seeds to generate different initial perimeters for them. These experimental results satisfy a normal distribution and Fig. 3 shows the statistical results. Then, we use paired T-test to conduct the significant test. The p-values for two sets of samples were less than the significance level of 0.05, which indicates the improvement of TaKE-SimplE is statistically significant compared with SimplE.

Generally, TaKE provides an effective method to utilize type features to extend existing KGE models. Types and entities benefit each other during embedding, producing promising results in the link prediction task.

### Ablation study

In order to study the effectiveness of the new type-constrained negative sampling strategy and relation-specific hyperplane mechanism, we conduct ablation studies on the dataset FB15K. First, we compare the link prediction performance between the original TaKE-ComplEx and its variant TaKE-ComplEx (unif), which uses uniform sampling instead of type-constrained sampling. Second, we further omit the relation-specific hyperplane mechanism RHM from TaKE-ComplEx(unif). The results of these comparisons are shown in Table 5. We can observe that the original TaKE-ComplEx using type-constrained sampling achieves better performance than TaKE-ComplEx(unif) employing uniform sampling. This result empirically demonstrates the effectiveness of the type-constrained negative sampling strategy. Furthermore, omitting the relation-specific hyperplane mechanism from TaKE-ComplEx(unif) makes it decline to TypeComplex, so it obtains comparable results with TypeComplex. This depicts that the ability to model type diversity is closely related to the performance of link prediction. Therefore, the relation-specific hyperplane mechanism plays a pivotal role in our approach.

**Table 5:** Ablation study of TaKE on FB15K.

Model	*MRR*	*Hits@1*	*Hits@3*	*Hits@10*
TaKE-ComplEx	**0.778**	**0.701**	**0.839**	**0.895**
TaKE-ComplEx(unif)	0.767	0.688	0.825	0.877
TaKE-ComplEx(unif)-RHM	0.760	0.684	0.815	0.868

Significant values are in bold.

**Figure 4:**
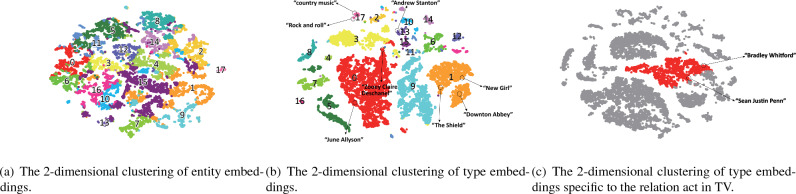
The visualization of entity and type embeddings clustering on FB15K.

### Entity/type embeddings clustering and visualization

We perform clustering of entity and type embeddings on FB15K, and visualize the clustering results to evaluate whether TaKE could capture type features. For this experiment, we first cluster entity and type embeddings produced by TaKE-SimplE using K-means^46^ (The superparameter K = 18) and then visualize them employing t-SNE^47^ to obtain 2-dimensional entity and type embeddings (originally including *d* and *k* dimensions). Different clusters are plotted with different colors to represent entity types. Figure 4a,b exhibit the clustering of entity embedding and type embedding after dimensionality reduction.

It is obvious that the clustering of type embeddings is compacter and separates type clusters better than the clustering of entity embeddings, which validates that type embeddings could collect type features better. We can also observe that some clusters of type embeddings are relatively independent, such as 1, 5 and 8, while others are close to each other or even overlap, such as 2, 11, 13, etc. That is because an entity may belong to multiple types, so it tends to locate at the intersection of multiple type clusters. Such as *Bradley Whitford*, who is not only an actor, but also a productor. While *Sean Justin Penn* is both an actor and a scriptwriter. But when they are connected with the relation *act in TV*, they all focus on the *actor* type. Our relation-specific projection method could further refine type embeddings to distinguish different type features of the same entity. As Fig. 4c shows, we project all type embeddings on the hyperplane of the relation *act in TV* and visualize them. The result shows that most actors and actresses including *Bradley Whitford* and *Sean Justin Penn* are grouped into the same cluster while others stay far away. This is because only entities that appear at the head position of the relation *act in TV* imply the *actor* type, while other entities do not. Our relation-specific projection method could capture this more fine-grained type feature specific to the relation *act in TV*, which demonstrates the effectiveness of the method.

## Conclusion and future work

In this paper, we propose a model-agnostic TaKE framework that could enhance traditional KGE models under no explicit type information supervision. By modeling type constraint and type diversity, our framework could capture and learn more-refined type features of each entity automatically. Besides, a new type-constrained negative sampling strategy is designed to flexibly incorporate prior knowledge of type constraint to construct high-quality negative samples for effective training. Experiments on four benchmark datasets for link prediction demonstrate that our framework is able to improve the performance of existing KGE models with lower time and space complexity. When combined with SimplE, TaKE could achieve state-of-the-art link prediction performance compared to all baselines. On the other hand, the clustering results of learned embeddings indicate that TaKE could capture type features and distinguish diverse types effectively.

In future work, we plan to incorporate temporal information into our framework for exploring time-aware KGE models and extend TaKE to the open-world assumption^48^.

## Data Availability

Some or all data, models, or code generated or used during the study are available from the corresponding author by request.
